# Experiences and Perceived Influence of the Artificial Intelligence–Based Health Education Accurately Linking System (AI-HEALS) on Health Behaviors Among Patients With Type 2 Diabetes: Qualitative Study

**DOI:** 10.2196/84605

**Published:** 2026-05-12

**Authors:** Jing Wang, Yibo Wu, Yang Jiang, Rantong Bao, Xinbao Gu, Bingyang Kong, Xinying Sun

**Affiliations:** 1 School of Public Health, Peking University, 38 Xueyuan Road, Haidian District, Beijing, 100191, China, 86 13691212050; 2 Department of Nursing, the Fourth Affiliated Hospital of School of Medicine, International School of Medicine, International Institutes of Medicine, Zhejiang University, Yiwu, China; 3 Jitang College, North China University of Science and Technology, Tangshan, China; 4 Center for Clinical Epidemiology Research, Affiliated Hospital of Inner Mongolia Medical University, Hohhot, China; 5 Capital Medical University, Beijing, China; 6 School of Traditional Chinese Medicine, Capital Medical University, Beijing, China

**Keywords:** artificial intelligence, type 2 diabetes, mobile health, self-management, qualitative research

## Abstract

**Background:**

The management of type 2 diabetes requires sustained self-management across diet, physical activity, medication adherence, and blood glucose monitoring; however, maintaining these behaviors in daily life remains difficult for many patients. Artificial intelligence–enabled and mobile health interventions have shown promise in supporting diabetes education and self-management, but evidence on how patients actually experience and use such systems in real-world primary care remains limited.

**Objective:**

This study aimed to explore patients’ experiences with the Artificial Intelligence–Based Health Education Accurately Linking System (AI-HEALS) and its perceived influence on self-management behaviors among patients with type 2 diabetes.

**Methods:**

This explanatory qualitative study was nested within the intervention arm of a cluster randomized controlled trial of AI-HEALS. Purposive maximum-variation sampling was used to recruit participants who varied by sex, age, diabetes duration, hemoglobin A_1c_ level, digital literacy, and level of platform use. Of the 25 patients approached from the intervention arm, 17 agreed to participate. Semistructured interviews were conducted 3 months after the intervention (August to December 2023) with participants recruited from 45 communities in the Daxing and Shunyi districts of Beijing, China. The qualitative component was designed to explain perceived mechanisms of behavior change, contextual facilitators and barriers, and implementation-related experiences not captured by trial outcomes alone. Interview transcripts were analyzed thematically in NVivo 12 by 2 independent researchers using consensus coding, an audit trail, and member checking.

**Results:**

Participants’ experiences with AI-HEALS were reflected in four themes: (1) catalyzing health awareness and concern, (2) empowering self-management practices, (3) navigating usability and engagement, and (4) enhancing psychological adaptation. Participants perceived that AI-HEALS made diabetes more visible in daily life through repeated reminders and accessible educational content; supported practical self-management decisions related to diet, physical activity, medication adherence, and glucose monitoring; and, in some cases, improved confidence while reducing uncertainty and diabetes-related stress. The findings also suggested meaningful variation in how the intervention was experienced, particularly in relation to prior illness experience, routine stability, social support, and digital confidence. Many participants mainly engaged with lower-burden features such as pushed articles and reminders, whereas more interactive functions, such as the chatbot, were used less often.

**Conclusions:**

Patients generally perceived AI-HEALS as a useful source of ongoing education, behavioral prompting, and everyday support for diabetes self-management. The findings suggest that artificial intelligence–enabled education may work not only by increasing knowledge but also by reinforcing awareness, translating guidance into feasible daily action, and supporting psychological adaptation. At the same time, the intervention’s perceived usefulness depended on whether its content was understandable, low burden, and compatible with users’ routines. These results support further refinement of literacy-sensitive, layered digital interventions and their integration into routine community-based primary care.

## Introduction

Diabetes, particularly type 2, has emerged as a significant global public health challenge. Over recent decades, the global diabetic population aged 20 to 79 years has surged to 537 million, with projections of 643 million by 2030 and 783 million by 2045. In China, the adult diabetes prevalence is notably high at 11.9%, affecting 140.9 million individuals, thereby significantly impacting quality of life and imposing a substantial health and economic burden on society [1,2]. Diabetes also elevates the risk of severe complications, including heart disease, stroke, kidney disease, retinopathy, and lower limb amputation [3,4]. Therefore, effective management strategies are urgently needed to address the rising number of patients and the diverse medical environments.

Traditional management approaches primarily focus on patient education, lifestyle modification, regular blood sugar monitoring, and medication adherence [5,6]. However, the complexity of daily insulin injections, medication management, inflexible lifestyles, and continuous health monitoring imposes cognitive burdens on patients, often leading to suboptimal outcomes [7]. Additionally, traditional management faces challenges such as patient nonadherence, underscoring the need for innovative solutions that simplify diabetes management and make it more accessible and effective for patients [8].

Mobile health (mHealth) technology leverages smartphones and wearables to deliver health information and services, enabling real-time patient monitoring and personalized communication with health care providers [9,10]. Systematic reviews and meta-analyses have consistently demonstrated that mHealth applications can significantly enhance blood glucose control and improve diabetes-related health behaviors in patients with type 2 diabetes mellitus (T2DM) [11,12]. When integrated with artificial intelligence (AI), these platforms can analyze large datasets to identify risk factors and deliver tailored interventions, thereby reducing the need for frequent face-to-face consultations and alleviating health care system burdens. Common features, such as reminder systems and incentives, have also been shown to improve treatment adherence [12].

AI—encompassing machine learning, natural language processing (NLP), and other technologies—aims to simulate human intelligence and is increasingly applied in diabetes care [13-15]. AI plays a crucial role in self-management by supporting blood glucose monitoring, dietary and exercise guidance, disease prediction, and personalized care [16-19]. The World Health Organization has recognized AI’s potential to enhance health literacy and patient engagement in chronic disease education. Several AI-driven health education systems have demonstrated clinical benefits: for example, the Smart AI-Enabled Diabetes system developed by Alotaibi et al [20] significantly reduced hemoglobin A1c (HbA_1c_) levels and improved diabetes knowledge in Saudi patients, whereas Chen et al [21] reported similar glycemic improvements using an mHealth-based education program for T2DM patients initiating insulin therapy.

This study used the Artificial Intelligence–Based Health Education Accurately Linking System (AI-HEALS), deployed on WeChat [22]. With over 1.2 billion monthly active users, WeChat’s integrated ecosystem—Mini Programs, Official Accounts, and payments—allows health management services to be embedded in everyday use, mitigating the retention and adherence problems common to standalone apps [22,23]. Its broad reach across urban and rural populations, including older adults, enhances access and equity; private group chats and privacy controls help safeguard sensitive health data [23]. Unlike Weibo’s primarily public broadcasting model, WeChat’s interaction-centric social communication fosters sustained peer support that facilitates behavior change. Leveraging this platform, AI-HEALS used low-cost, widely available mobile communication to deliver continuous, convenient diabetes management directly to patients’ homes.

Based on the above, this qualitative study aims to answer the following research questions: (1) How do patients with T2DM perceive the influence of AI-HEALS on their self-management behaviors (diet, physical activity, medication adherence, and glucose monitoring)? (2) What are patients’ experiences with the usability and acceptability of the AI-HEALS system? (3) What facilitators and barriers to behavior change do patients encounter when using AI-HEALS, and how do features such as personalized reminders and educational content delivery shape these experiences?

## Methods

### Study Design and Trial Context

This study was designed as an explanatory qualitative study nested within the intervention arm of a cluster randomized controlled trial. It was intended to clarify perceived mechanisms of behavior change, contextual influences on use, and implementation-related experiences that were not captured by trial outcomes alone. The parent trial, registered at the Chinese Clinical Trial Registry (ChiCTR2300068952; registered on February 3, 2023), aimed to evaluate the effectiveness of AI-HEALS on glycemic control and self-management behaviors. In contrast, this qualitative substudy was designed to address questions that quantitative outcomes alone could not answer: how participants perceived the intervention to influence daily self-management, which contextual factors facilitated or constrained its use, and which implementation-related features shaped engagement in community-based primary care. Thus, the qualitative component was not intended to retest efficacy but to explain perceived mechanisms, user experiences, and real-world delivery processes within the intervention arm. The trial protocol has been published elsewhere [24].

### Study Setting and Participants of the Parent Trial

The parent trial was conducted from August to December 2023 across 45 community health centers in the Daxing and Shunyi districts of Beijing. A total of 406 participants were enrolled using cluster sampling, with each community health center recruiting 8 to 10 patients.

- Inclusion criteria for the trial were residents aged 18 to 75 years with a diagnosis of T2DM (fasting plasma glucose ≥7 mmol/L, 2-h plasma glucose ≥11.1 mmol/L, or HbA_1c_ ≥6.5%); permanent residency in Beijing; ability to use smartphones and WeChat; willingness to adhere to the study; and not participating in other studies.
- Exclusion criteria were type 1 diabetes, gestational or secondary diabetes; severe diabetes complications; recent radiotherapy or chemotherapy; severe intellectual disability, Alzheimer disease, or other mental disorders; or prior use of psychotropic drugs.

Clusters were randomized 1:1 using a computer-generated sequence, assigning 208 participants to the intervention group (standard diabetes primary care plus AI-HEALS) and 198 participants to the control group (standard diabetes primary care alone). Complete blinding of participants and researchers was not feasible due to the nature of the intervention.

### Qualitative Substudy Sampling and Recruitment

For this qualitative substudy, a purposive maximum variation sampling strategy was used to select information-rich cases from the intervention arm. Sampling criteria were designed to capture a broad range of experiences and perspectives, including sex (male/female), age (<60 y/≥60 y), diabetes duration (<5 y/5–10 y/>10 y), HbA_1c_ levels (<7%/≥7%), self-reported digital literacy (high/moderate/low), and level of platform use (frequent/moderate/infrequent based on login frequency and feature usage).

From the 208 participants in the intervention arm, we purposively approached 25 individuals who represented diverse combinations of the sampling criteria. Recruitment was conducted via telephone by a trained research assistant who explained the purpose of the qualitative interview and invited participation. Of the 25 individuals approached, 17 agreed to participate, yielding a response rate of 68%. Reasons for nonparticipation among the 8 decliners included lack of interest (n=3, 38%), time constraints due to work or family obligations (n=3, 38%), and concerns about privacy (n=2, 25%).

The sample size was determined by data saturation, defined as the point at which no new themes emerged from subsequent interviews. Saturation was achieved after 17 interviews, with no new themes emerging from the final 2 interviews. The final sample consisted of 17 AI-HEALS users from the intervention arm.

### Introduction to the AI-HEALS

The intervention was delivered through a 2-layer architecture: the backend AI-HEALS and the frontend “Beiyi Sugar Manager” WeChat Mini Program (Tencent Holdings Ltd.). AI-HEALS used a hybrid logic architecture rather than a fully generative model. Specifically, the conversational function relied on vendor-provided NLP and natural language understanding modules to identify user intent and extract key terms, after which the system retrieved preconfigured responses from a curated diabetes education knowledge base. In parallel, reminder delivery and content recommendation were governed primarily by deterministic rule-based logic using baseline patient profile variables (eg, age, duration of diabetes, HbA_1c_ level, and self-reported digital literacy), recent article reading history, manual health logs, and regimen-related schedules. This architecture allowed the system to combine conversational access with stable, auditable delivery of education and reminders.

The “Beiyi Sugar Manager” Mini Program was the patient-facing application embedded within the WeChat ecosystem, through which participants accessed all intervention components without installing a standalone app. All communications—including educational content, AI chatbot interactions, automated reminders, and message notifications—were delivered via the WeChat Mini Program interface.

Educational messages were prioritized according to broad self-management domains (diet, exercise, medication, monitoring, complications) and adjusted using recent engagement patterns, such as categories previously read, reading duration, and frequency of health data entry. Reminder schedules were anchored to user-entered medication and monitoring routines and could be intensified when recent logging was irregular. The educational knowledge base and decision rules were developed by the research team with clinical and public health input before trial deployment. Because the dialog platform was commercially provided, the underlying training corpus, model weights, and proprietary internal validation metrics were not available to the research team for publication.

**Figure 1.**
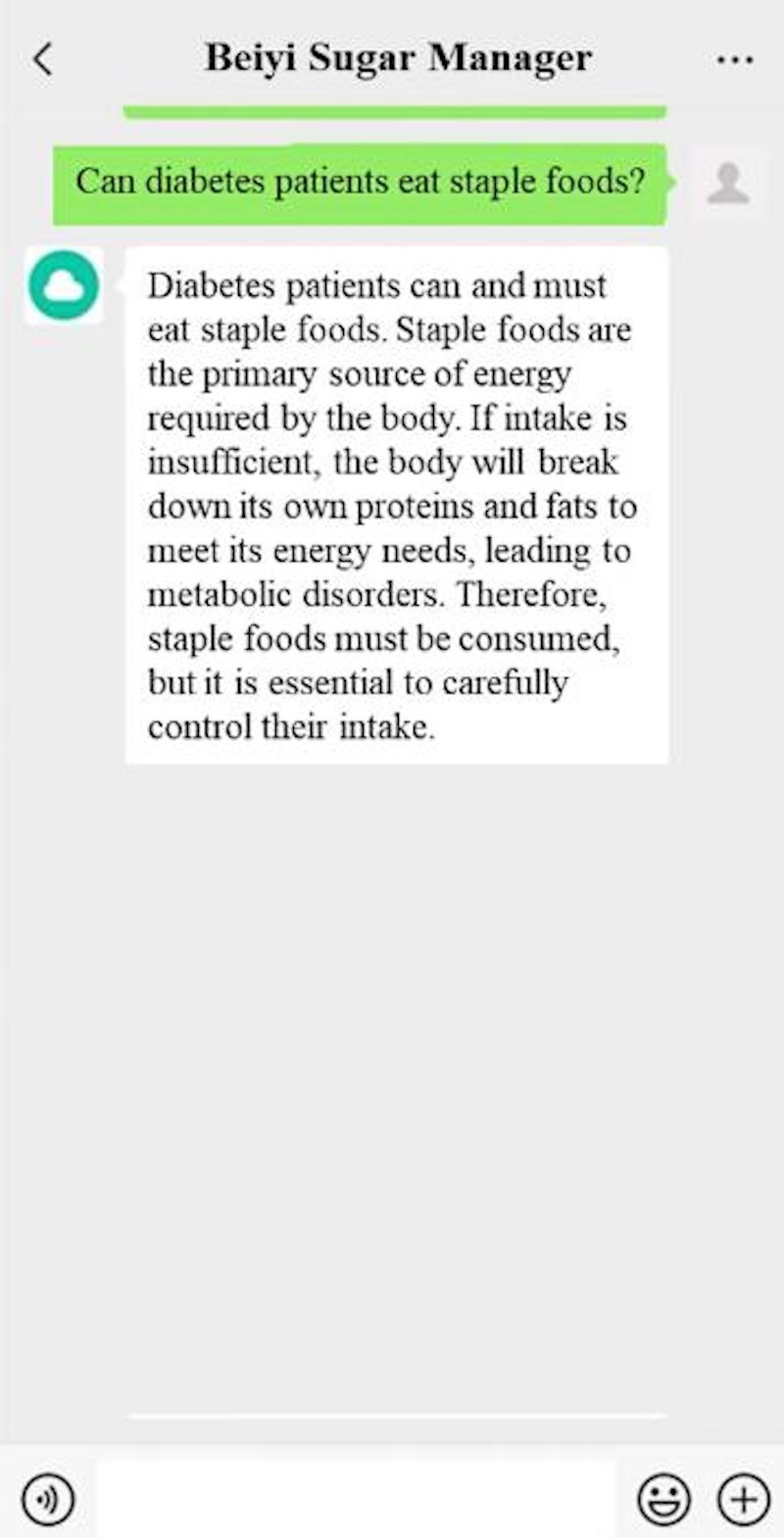
Interaction with the artificial intelligence chatbot in the “Beiyi Sugar Manager” mini program. The screenshot illustrates a participant querying about dietary recommendations and receiving a personalized response based on the system’s knowledge base. Users initiate conversations by typing questions, and the chatbot provides evidence-based information tailored to individual profiles.

**Figure 2.**
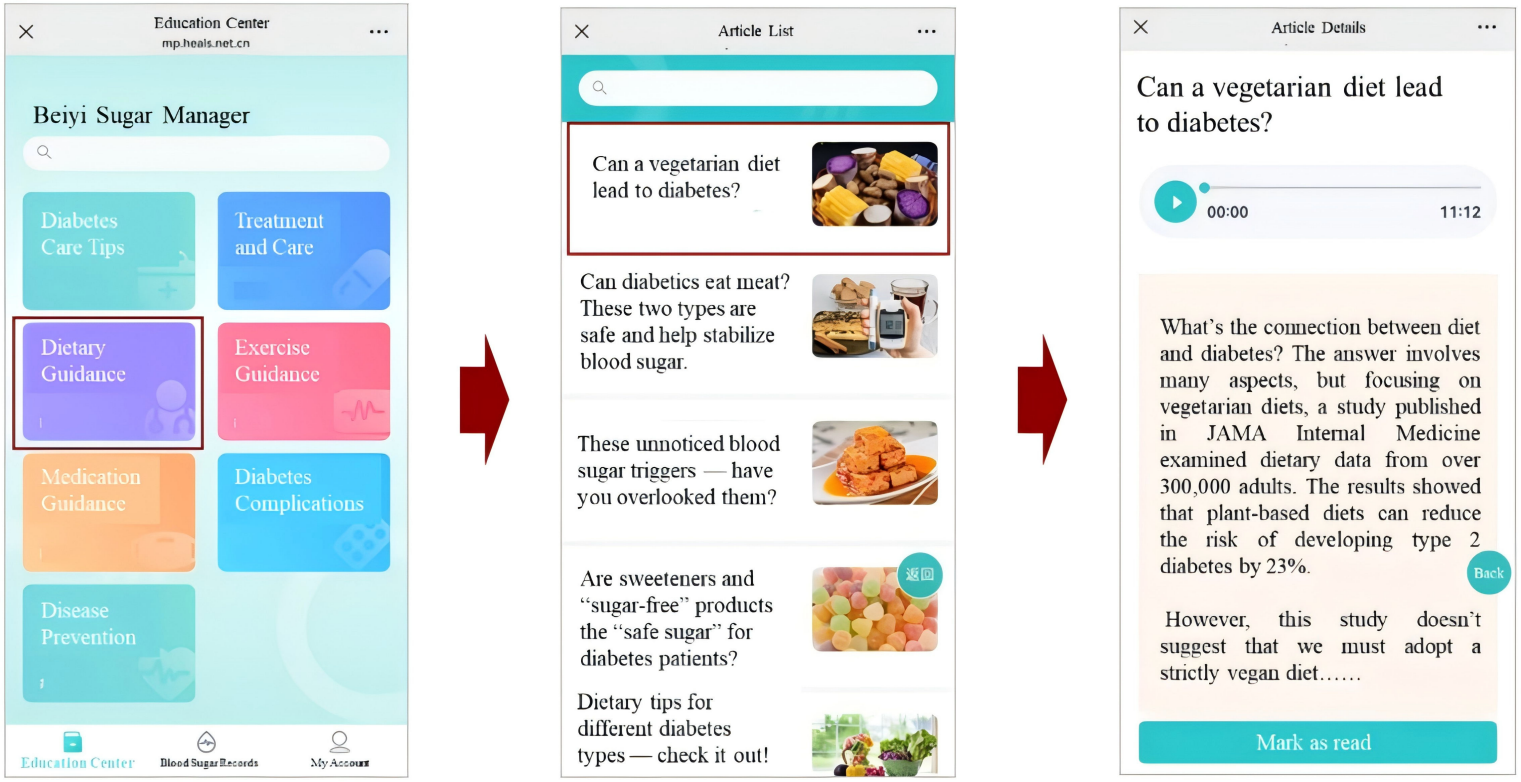
Educational content delivery and user engagement features. The panel shows the “Education Center” interface with categorized articles (eg, dietary guidance, exercise guidance).

**Figure 3.**
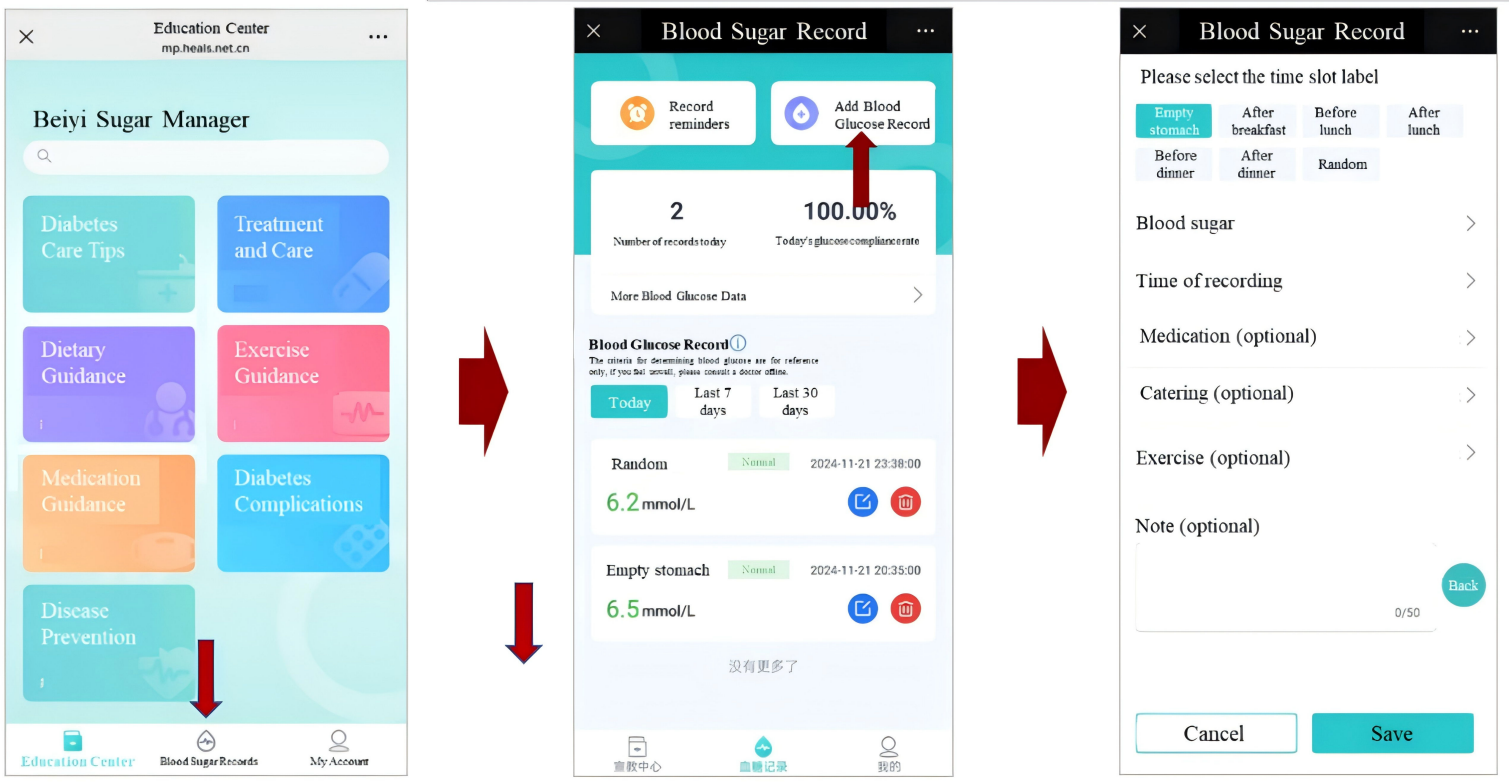
Blood glucose monitoring and recording interface. Users can log their blood glucose values (fasting, postprandial) and view trends over time. The system provides feedback on readings and integrates this data with reminder functions.

**Figure 4.**
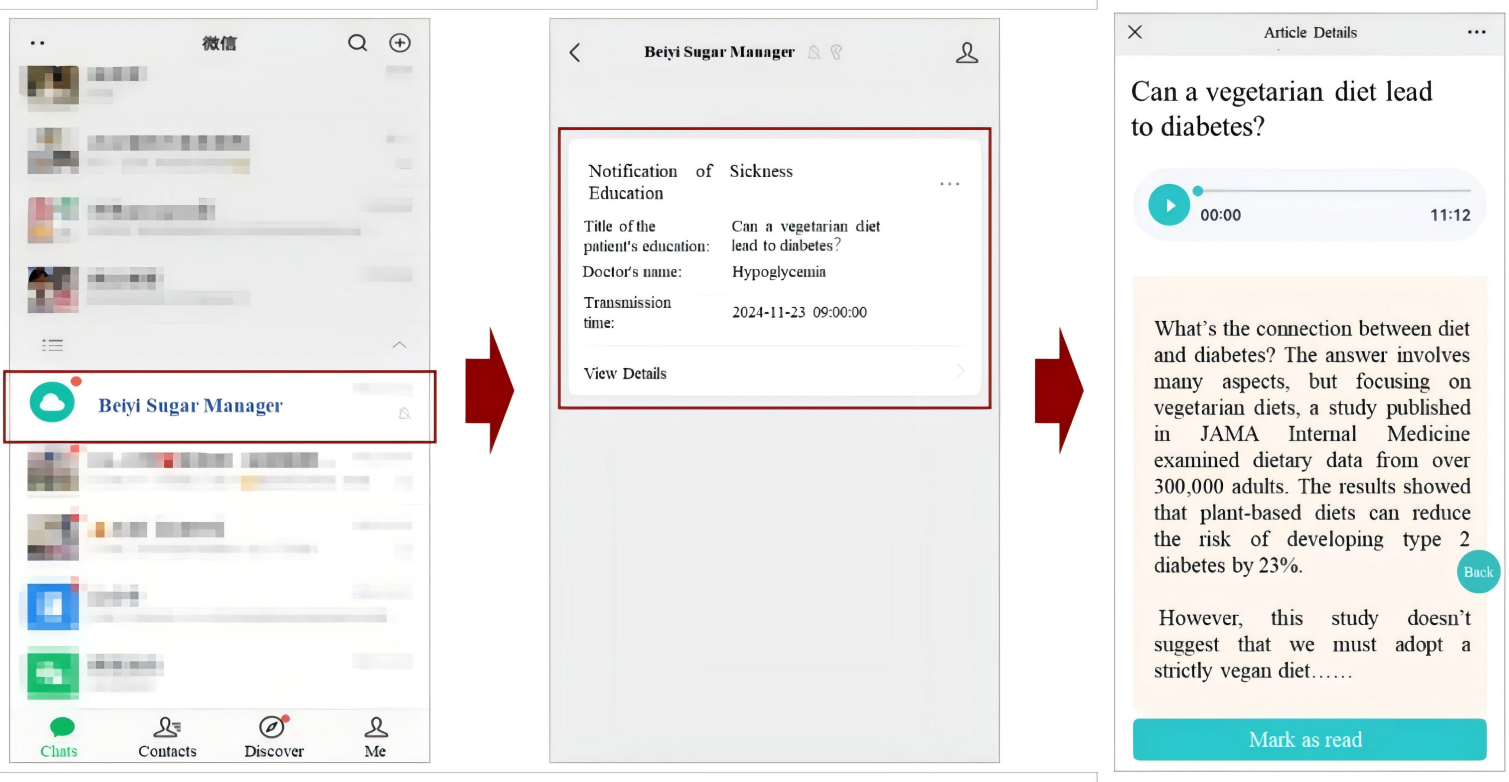
Automated reminder system for medication and monitoring. Participants receive scheduled reminders through the WeChat mini program interface, similar to receiving messages from a contact. Reminders are personalized based on individual medication regimens and monitoring schedules.

**Figure 5.**
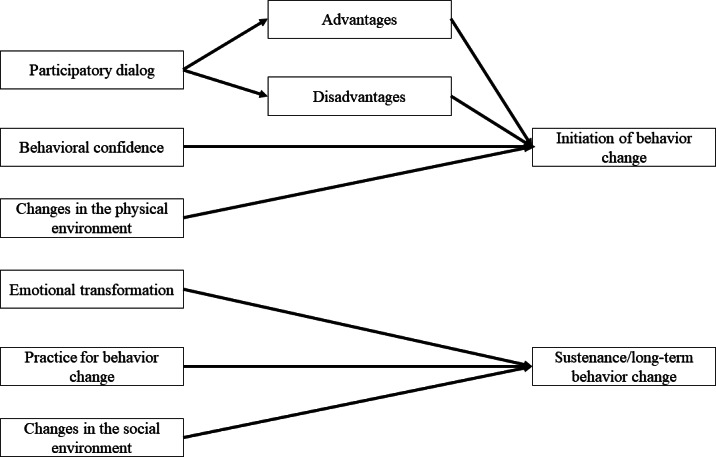
Multi-Theory Model (MTM) of health behavior change framework. The model illustrates the 2-stage process of behavior change (initiation and maintenance) and the theoretical constructs (eg, participatory dialog, behavioral confidence, changes in the physical environment) that informed the intervention design.

The mini program provides 4 main functional modules powered by the AI-HEALS backend:

1. AI-powered conversational agent (chatbot).

- Frontend (patient view): Within the “Beiyi Sugar Manager” Mini Program, participants can access an AI chatbot by typing questions about T2DM diet, exercise, and medication management. The chatbot provides answers in a conversational format, offering immediate responses to user queries within the scope of the system’s diabetes knowledge base (Figure 1).

- Backend (AI-HEALS support): This functionality is powered by the Emotibot-produced Bot Factory dialog AI platform (ZhuJian Intelligence Technology) [25]. The platform uses NLP and natural language understanding algorithms to analyze participant queries, extract contextual information, and retrieve the most relevant responses from the knowledge base. Responses are retrieved and prioritized using user profile attributes (age, duration of diabetes, HbA_1c_ level, and digital literacy) together with prior interactions, enabling tailored delivery of health education within the intervention context.

2. Personalized educational content library.

- Frontend (patient view): The mini program features an “Education Center” that provides participants with access to a structured library of educational articles (Figure 2). Content is categorized into 7 areas, including “Dietary Guidance” (23 articles), “Exercise Guidance” (17 articles), “Medication Guidance” (18 articles), “Diabetes Complications” (9 articles), and “Disease Prevention” (23 articles). Articles are pushed to users based on their reading history and preferences.
- Backend (AI-HEALS support): The AI-HEALS backend tracks user activity—including number of articles read, duration, and categories of interest—and applies a rule-based personalization algorithm. Educational content is pushed 1 to 3 times per week according to user preferences, engagement patterns, and theoretically grounded behavior-change principles, ensuring both relevance and reinforcement of self-management behaviors.

3. Physiological monitoring and reminder system.

- Frontend (patient view): The mini program allows participants to manually log key physiological indicators, such as blood glucose levels (fasting and postprandial) and blood pressure (Figure 3). It also delivers automated reminders for medication intake and blood glucose checks directly through the WeChat interface, appearing similarly to messages from a contact.
- Backend (AI-HEALS support): The reminder scheduling engine uses logged health data and prescribed medication regimens to generate individualized notifications. Logged health data are used to adjust reminder delivery, for example, by increasing reminder frequency when recent logging is inconsistent or when readings suggest a need for closer monitoring.

4. Theory-driven automated messaging.

- Frontend (patient view): Participants receive 1 to 3 automated messages per week with tips and information related to T2DM self-management, supporting both the initiation and maintenance of healthy behaviors (Figure 4). These messages are delivered through the “Beiyi Sugar Manager” Mini Program.
- Backend (AI-HEALS support): The content and timing of these automated messages are theoretically grounded in the Multi-Theory Model (MTM) of health behavior change (Figure 5) [26]. The backend system operationalizes the MTM framework by delivering content tailored to support users through the stages of behavior initiation (eg, starting an exercise routine) and behavior maintenance (eg, sustaining long-term dietary changes). Messages are selected from the “Education Center” library, and message selection accounts for prior engagement, health metrics, and interaction history, enabling rule-guided tailoring to individual user needs within the intervention.

### Data Collection and Analysis

Semistructured interviews were conducted with all 17 participants 3 months after the intervention to explore their experiences and perceived influence of AI-HEALS on self-management behaviors, including diet, physical activity, and continuous glucose monitoring. Interviews, lasting 30 to 60 minutes, were held either in person or by telephone. Participants were encouraged to recount context-specific scenarios and critical incidents, describe their interactions with the system and challenges encountered, and propose suggestions for improvement related to behavior change. All interviews were audio recorded with permission and transcribed verbatim.

Thematic analysis of the transcripts was performed using NVivo software (version 12; QSR International). To enhance transparency and reproducibility, an audit trail of coding decisions was maintained. Two researchers (JW and YJ) independently coded the transcripts. After initial coding of the first 3 transcripts, the research team discussed the preliminary codebook and resolved any discrepancies through consensus; subsequent consensus meetings were held to refine codes and develop themes. The coding process was primarily inductive, allowing themes to emerge from the data. Following the completion of initial thematic analysis, member checking was conducted with 5 participants, who were asked to review a summary of the findings and provide feedback on whether the interpretations resonated with their experiences. This feedback confirmed the credibility of the themes and did not necessitate substantial revisions.

### Ethical Considerations

This study was approved by the Biomedical Ethics Committee of Peking University (IRB00001052-22058; approved on June 6, 2022) and registered with the Chinese Clinical Trial Registry (ChiCTR2300068952; registered on February 3, 2023). Written informed consent was obtained from all interview participants before data collection. Participants did not receive financial compensation for participation. Several measures were implemented to protect participant anonymity and data security within the WeChat environment. All data transmitted through the “Beiyi Sugar Manager” mini program were encrypted using industry-standard protocols. Participant identifiers were replaced with unique study codes, and data were stored on secure servers accessible only to authorized research personnel. Participants were informed that while WeChat’s platform has its own privacy policy, the research team implemented additional safeguards, including not collecting WeChat IDs or contact lists and advising participants not to share personal health information through unsecured channels. The mini program was designed to comply with China’s Personal Information Protection Law and relevant data security regulations.

## Results

### Patient Demographics

Demographic characteristics of the patients are shown in Table 1.

**Table 1.** Patient demographics.

Variables	Total (N=17)^a^
BMI, mean (SD)	26.33 (3.60)
Waist circumference, mean (SD)	97.88 (13.45)
Sex, n (%)	
Female	6 (35.29)
Male	10 (58.82)
Unknown	1 (5.88)
Ethnicity, n (%)	
Han	16 (94.12)
Unknown	1 (5.88)
Income (CNY)^b^, n (%)	
＞9000	4 (23.53)
≤3000	4 (23.53)
3001‐9000	8 (47.06)
Unknown	1 (5.88)
Occupation status, n (%)	
Employed	7 (41.18)
No fixed occupation/freelance	2 (11.76)
Retired	5 (29.41)
Unemployed	2 (11.76)
Unknown	1 (5.88)
Marital status, n (%)	
Married	16 (94.12)
Unknown	1 (5.88)
Hukou, n (%)	
Agricultural	7 (41.18)
Nonagricultural	9 (52.94)
Unknown	1 (5.88)
Current health insurance, n (%)	
Beijing medical insurance	15 (88.24)
No medical insurance	1 (5.88)
Unknown	1 (5.88)
People living with you in the past 2 months, n (%)	
Living alone	1 (5.88)
Living with family members	15 (88.24)
Unknown	1 (5.88)
Education, n (%)	
Bachelor’s degree	1 (5.88)
Junior college	3 (17.65)
Junior high school	6 (35.29)
Secondary vocational school	1 (5.88)
Senior high school	5 (29.41)
Unknown	1 (5.88)
Occupation, n (%)	
Agricultural/forestry/animal husbandry/fishery worker	1 (5.88)
Designer/design professional	1 (5.88)
Finance/accounting/cashier/audit	1 (5.88)
Freelancer	2 (11.76)
Full-time homemaker	1 (5.88)
Industrial/manual worker	1 (5.88)
Marketing/sales/business	1 (5.88)
Medical/health care staff	1 (5.88)
Product/operations staff	1 (5.88)
Retired	2 (11.76)
Service industry staff	3 (17.65)
Technical developer/engineer	1 (5.88)
Unknown	1 (5.88)
Type of health insurance, n (%)	
Government-funded medical care	1 (5.88)
New rural cooperative medical scheme	4 (23.53)
No medical insurance	1 (5.88)
Unknown	1 (5.88)
Urban employee basic medical insurance	6 (35.29)
Urban resident basic medical insurance	4 (23.53)
Any other household members with diabetes, n (%)	
Father	1 (5.88)
Father and siblings	1 (5.88)
Mother	2 (11.76)
Mother and siblings	1 (5.88)
None	7 (41.18)
Siblings	3 (17.65)
Unknown	2 (11.76)
Duration since diabetes diagnosis, n (%)	
1 year	1 (5.88)
2 years	1 (5.88)
3 years	1 (5.88)
10 years	4 (23.53)
12 years	2 (11.76)
20 years	1 (5.88)
Unknown	7 (41.18)

a Information for 1 participant (Q) was missing from the dataset; they are included in the “Unknown” category where applicable.

b Chinese yuan; an exchange rate of US $1=CNY ¥6.906 is applicable.

The study included 17 participants with T2DM, with a mean BMI of 26.33 (SD 3.60)  kg/m² and a mean waist circumference of 97.88 (SD 13.45) cm. The majority were male (n=10, 58.82%) and of Han ethnicity (n=16, 94.12%), with approximately half reporting a monthly income of 3001 to 9000 CNY (based on an exchange rate of US $1=CN ¥6.906). Participants varied in occupation and education, with 41.18% (n=7) employed, 29.41% (n=5) retired, and educational attainment ranging from junior high school to a bachelor’s degree. Most participants were covered by Beijing medical insurance (n=15, 88.24%) and lived with family members, while 47.06% (n=8) reported a family history of diabetes. The duration since diagnosis ranged from 1 to 20 years, with 41.18% (n=7) of participants reporting it as unknown.

A comprehensive overview of each participant’s demographic and health-related information is available in Multimedia Appendix 1.

### Qualitative Findings

Participants accessed the AI-HEALS intervention via the “Beiyi Sugar Manager” WeChat Mini Program (hereafter referred to as “the mini program”). Four main themes emerged from the analysis (Table 2), each with several subthemes, illustrating the perceived impact of the AI-HEALS intervention on participants’ self-management journeys. Below, each theme and subtheme is described with illustrative quotations to demonstrate how the interpretation was grounded in participants’ accounts.

**Table 2.** Summary of qualitative themes, subthemes, and illustrative quotations.

Theme and subtheme	Sample quotations from interviews
Catalyzing health awareness and concern	
Deepened understanding of complications.	Patient D: “Complications are quite serious… like ‘three more and one less,’ foot ulcers, skin itching, or blindness. I’ve had my eye fundus checked, no bleeding… I usually pay attention to my diet to avoid large blood sugar fluctuations, because high blood sugar can damage blood vessels and form plaques, which is bad for the brain and body.”
	Patient N: “I mainly worry about the heart, eyes, and feet. My eyes are sometimes blurry; I’m most afraid of bleeding in the eye fundus… It’s very serious, especially the eyes. Sometimes I worry about going blind.”
Reinforcing the importance of self-management.	Patient D: “To live a long life and maintain a good quality of life, you must have the confidence and perseverance to persist in controlling your blood sugar. You can’t ignore your diet, especially during the holidays. The saying ’illness enters by the mouth’ is very true.”
	Patient F (on using the mini-program): “It definitely [helped], it gave me that awareness in my mind… I pay more attention now.”
Empowering self-management practices	
Dietary improvements.	Patient B: “Before, I thought anything with sugar was off-limits, I wouldn’t even eat fruit. Now, after reading the mini-program, I can judge which foods are lower in sugar and can be eaten in moderation… I know some fruits, like firmer apples, aren’t that high in sugar.”
	Patient O: “The dietary structure has changed. [the program] tells you what to eat and how to eat it.”
	Patient K: “There are things I want to eat, but now I control the amount. I’m not so casual about it anymore.”
Enhanced physical activity.	Patient I: “There’s been improvement. Before, I forced myself to walk over 10,000 steps a day… But the doctor [via the program] suggested that based on my age, it’s better to limit it to 5000 to 7000 steps. Now my walks are more relaxed, about half an hour, mainly strolling instead of forced exercise.”
	Patient P: “When the weather is bad, I do half an hour of activity at home after meals. Stand when you can, sit instead of lie down. I do some arm and leg stretches…”
	Patient A: “My exercise time has increased. Everyone cares about their health; [the program] helps not only with blood sugar but also with blood lipids.”
Improved adherence to medication and monitoring.	Patient H: “The blood sugar testing and uploading function… it’s very convenient. Yes, it’s really helpful for my health.”
	Patient P: “I definitely follow the reminders. I have to take my medication three times a day with meals. Even if I don’t eat, I still have to take it, otherwise I might get hypoglycemia.”
	Patient N: “[The mini-program] at least reminds me today to eat less of the foods that will spike my blood sugar. It works. If it didn’t remind me, I wouldn’t know, and it wouldn’t help.”
Navigating usability and engagement	
Primary engagement with passive features.	Patient A: “I mainly just read the popular articles.”
	Patient L: “I mainly look at the articles that are sent.”
	Patient Q: “I basically don’t use other functions; I mainly just read the articles.”
Variable engagement and digital literacy.	Patient K: “I don’t know how to do the other things. Sometimes I just open it and look at the articles… I can understand the simple ones, but the complicated ones I can’t.”
	Patient C: “For someone like me with a simple, regular life, I basically don’t need the reminders. But for younger patients, this kind of reminder would be very useful.”
Enhancing psychological adaptation	
Fostering self-efficacy and confidence.	Patient D: “I have the confidence and will to control my blood sugar.”
	Patient H: “One hundred percent!” (when asked about his confidence to continue).
	Patient Q: “I definitely have the confidence, and the will is strong. Everyone wants to live. Now, I can manage my situation on my own, without relying on others.”
Alleviating anxiety and distress.	Patient H: “It [the mini-program] helps, I pay much more attention now. Before I used it, I didn’t know these basic facts. Now I’ve learned them through the mini-program.”

#### Theme 1: Catalyzing Health Awareness and Concern

This theme captures participants’ experiences with the intervention, particularly how its educational content and reminders shaped their understanding of diabetes and its potential consequences and how it influenced their motivation for self-management.

##### Subtheme 1.1: Deepened Understanding of Complications

Many participants reported that the information provided through the mini program increased their awareness of the serious long-term complications of diabetes, such as retinopathy, cardiovascular disease, and diabetic foot. For example, Patient D described complications as “quite serious,” whereas Patient N said she was “most afraid of bleeding in the eye fundus” and “going blind.” This heightened perceived risk underscored the importance of proactive management.

##### Subtheme 1.2: Reinforcing the Importance of Self-Management

Beyond knowledge, the system’s regular prompts and accessible information served as a constant reminder of the need for vigilance, reinforcing participants’ commitment to their self-management routines. Patient D emphasized that, to maintain a good quality of life, one must persist in controlling blood sugar, and Patient F noted that the mini program “gave me that awareness in my mind,” making her “pay more attention now.”

### Theme 2: Empowering Self-Management Practices

This theme reflects participants’ experiences in translating increased awareness into behavioral changes, with the mini program serving as a supportive tool.

#### Subtheme 2.1: Dietary Improvements

The most frequently cited area of change was diet. Participants reported that the educational articles helped them distinguish between healthier and less healthy food choices, understand portion sizes, and adopt more flexible but better-informed eating habits. Patient B, for example, explained that after using the mini program, he no longer believed that all fruit was forbidden and felt better able to judge which foods could be eaten in moderation.

#### Subtheme 2.2: Enhanced Physical Activity

The mini program also influenced participants’ exercise habits. Some reported becoming more consistent, whereas others adjusted their routines based on the guidance received. Patient I described shifting from a forced goal of more than 10,000 steps per day to more age-appropriate walking, whereas Patient P reported doing indoor activity after meals when the weather was poor.

#### Subtheme 2.3: Improved Adherence to Medication and Monitoring

The reminder functions were highlighted as a key facilitator for medication adherence and regular blood glucose monitoring, particularly for those with less structured daily routines. Patient H described the testing and uploading function as “very convenient,” and Patient P said she “definitely” followed the medication reminders.

### Theme 3: Navigating Usability and Engagement

This theme explores how participants interacted with the technology itself, revealing patterns of use, preferences for certain features, and barriers to full engagement.

#### Subtheme 3.1: Primary Engagement With Passive Features

Most participants reported primarily using lower-burden features of the system, especially pushed educational articles and reminders, rather than more active functions. As Patients A, L, and Q each noted in different ways, they mainly read the articles that were sent and rarely used other modules.

#### Subtheme 3.2: Variable Engagement and Digital Literacy

Engagement levels appeared closely linked to digital literacy and personal preference. Patient K stated that he could understand “the simple ones” but not the “complicated ones,” whereas Patient C felt that reminders were less necessary because his daily routine was already highly structured. Together, these accounts suggest that lower engagement with some functions did not necessarily mean low perceived usefulness.

### Theme 4: Enhancing Psychological Adaptation

Beyond practical behavior change, participants described a positive psychological impact from using the system, which contributed to a greater sense of control and a reduced emotional burden.

#### Subtheme 4.1: Fostering Self-Efficacy and Confidence

Successfully implementing the information and reminders from the mini program appeared to build participants’ confidence in their ability to manage their condition effectively. Patient D said she had the “confidence and will” to control her blood sugar, and Patient Q similarly reported feeling able to manage the condition “on my own, without relying on others.”

#### Subtheme 4.2: Alleviating Anxiety and Distress

For some participants, the knowledge and structure provided by the system helped mitigate anxiety and distress associated with diabetes. Patient H said that learning basic facts through the mini program reduced uncertainty, and Patient C described the clear guidance on diet and medication as reassuring.

## Discussion

This qualitative study explored the experiences and the perceived influence of AI-HEALS on self-management behaviors among 17 patients with type 2 diabetes. Four overarching themes emerged: catalyzing health awareness and concern, empowering self-management practices, navigating usability and engagement, and enhancing psychological adaptation. Together, these findings suggest that an AI-enabled, theory-informed mHealth intervention may support self-management not only by delivering information but also by embedding reminders, guidance, and reinforcement into everyday routines. At the same time, participants’ accounts indicate that the intervention did not function in exactly the same way for all users and that these differences were closely related to prior illness experience, routine stability, social support, and digital confidence.

### Catalyzing Health Awareness and the Knowledge-Behavior Gap

Participants’ reports that AI-HEALS deepened their understanding of diabetes complications align with the literature showing that knowledge is an important but often insufficient condition for behavior change in diabetes self-management [27,28]. In this study, awareness often extended beyond general education and became tied to concrete concerns about blindness, cardiovascular disease, and diabetic foot. This suggests that the intervention helped translate abstract risk into a more personally meaningful understanding.

The findings also indicate that the value of AI-HEALS lay not only in transmitting information but also in sustaining attention to diabetes in daily life. Repeated prompts and accessible educational content appeared to keep the condition salient over time, thereby helping to address the knowledge-behavior gap described in previous diabetes education research [29]. In this respect, AI-HEALS differs from more conventional education delivered in discrete sessions [5,6] by providing ongoing reinforcement. This pattern is also consistent with the MTM, particularly its emphasis on participatory dialog as a motivational process in behavior change [26].

At the same time, the nature of this awareness appeared to vary across participants. For individuals with a longer duration of diabetes, such as Patients D and N, the intervention seemed to reinforce and sharpen existing illness understanding, although the content of that awareness differed: one account reflected a broader physiological understanding of vascular risk, whereas another focused more strongly on feared complications such as blindness. By contrast, for a newly diagnosed participant such as Patient O, the intervention appeared to play a more foundational role in shaping initial self-management understanding. This pattern suggests that AI-enabled education may function differently across the diabetes trajectory, serving as reinforcement for some users and as initial structuring support for others. Family context may also have influenced how risk information was received, as prior household exposure to diabetes could heighten vigilance toward complications and self-management [30].

### Empowering Self-Management: Beyond Generic Recommendations

The sense of empowerment described by participants resonates with Anderson and Funnell [31] patient empowerment model, which emphasizes active patient ownership of diabetes management. In this study, the perceived value of AI-HEALS lay not simply in offering recommendations but also in helping participants translate broad self-management principles into concrete, day-to-day decisions.

Dietary change was the most frequently discussed area of improvement, which is consistent with systematic reviews identifying nutrition as one of the most responsive domains in diabetes self-management interventions [10,11]. Participants’ accounts suggested a movement away from rigid restriction toward more flexible and informed judgment, a pattern broadly in line with work on personalized nutrition and tailored self-management support [19]. Importantly, however, these changes did not appear uniform across users. For newly diagnosed participants, dietary guidance seemed to support the establishment of initial management routines. For others with more experience, it appeared to refine existing practices rather than create them from scratch. These differences suggest that the same digital intervention may support both habit formation and habit adjustment, depending on patients’ prior self-management history.

The findings on physical activity similarly indicate that participants valued guidance not just when it encouraged “more exercise” but also when it made activity more appropriate, feasible, and compatible with daily life. The accounts of individualized walking targets and indoor alternatives suggest that users valued optimized guidance over generic behavioral goals. This is in line with emerging evidence that, for adults managing chronic disease, the appropriateness of activity may matter as much as quantity [17]. The case of Patient P also suggests that such support may be particularly relevant for users who manage much of their care independently in everyday life.

At the same time, the findings point to an important tension between personalization and accessibility. Tailored guidance was clearly valued, but some participants also indicated difficulty understanding more complex content. This was especially evident in the account of a participant with lower educational attainment who remained engaged with simpler materials while avoiding more complicated ones. This suggests that digital support may be less effective when information is clinically relevant but insufficiently accessible to users with lower literacy or lower digital confidence. For intervention design, these findings support the importance of tailoring that is not only behaviorally targeted but also sensitive to comprehension and usability.

### Navigating Usability and Engagement in Everyday Use

A notable finding was that many participants mainly engaged with lower-burden features, such as pushed articles and reminders, whereas more active functions, including the chatbot, were used less often. This pattern aligns with international research on digital diabetes interventions, which frequently report challenges in sustaining engagement and note that usage intensity does not necessarily correspond directly to perceived benefit [28,32].

In the present study, passive engagement still appeared meaningful. For some participants, reading articles and receiving reminders was sufficient to support awareness, routine, and behavioral adjustment. This pattern may reflect the effort-reward balance described in prior health technology research, whereby users gravitate toward features that provide clear benefits with minimal cognitive burden [33]. It may also suggest that interactive components, such as chatbots, need to demonstrate clearer added value if they are to become central components of engagement.

Participants’ accounts further indicate that engagement was shaped by the interaction of digital confidence, education, prior routines, and perceived need. The contrast between a participant with lower educational attainment who found “complicated” features difficult and a highly educated participant who used the system selectively because his daily life was already structured suggests that low use does not necessarily mean low usefulness. Instead, selective engagement may reflect different routes to perceived benefit. These findings complicate a simple digital divide narrative: age alone did not appear sufficient to explain engagement, and educational background, routine stability, and prior exposure to structured work or technology may have been equally important in shaping how participants used the intervention.

In addition, the continued engagement reported by several lower-income participants supports the practical value of delivering AI-HEALS through WeChat. By relying on an already familiar platform rather than a standalone app, the intervention may have reduced access barriers associated with downloading, registration, or cost. In this respect, the platform choice itself may have contributed to usability and equity in engagement.

### Psychological Adaptation: The Less Visible Dimension of Digital Support

The psychological benefits described by participants—greater confidence, reassurance, and reduced uncertainty—highlight an important dimension of digital self-management support that is often less visible in studies focused primarily on clinical outcomes. These accounts are consistent with the literature emphasizing the emotional burden of diabetes and the value of supportive interventions for coping and self-management [34,35].

Importantly, the perceived psychological benefit of AI-HEALS appeared to arise indirectly through better understanding, clearer routines, and more manageable daily tasks rather than through explicit psychological counseling. Participants described feeling less overwhelmed and more capable when management tasks became more understandable and structured. This suggests that psychological adaptation may be closely intertwined with practical self-management support rather than standing apart from it. In this sense, the intervention may have reduced resistance to the burden of ongoing self-management by transforming diabetes from an overwhelming threat into a more manageable set of daily tasks [36].

This pattern can also be related to the Health Action Process Approach, which distinguishes motivational processes from the volitional support needed to sustain action [37-39]. In this study, educational content appeared to strengthen motivation by reinforcing awareness of risk and outcomes, while reminders and monitoring functions appeared to support the volitional work of action planning, routine maintenance, and coping. The coexistence of behavioral change and reduced uncertainty in participants’ accounts is consistent with this dual-support process.

The findings also suggest that psychological adaptation varied according to illness experience and everyday context. For participants with more established self-management histories, confidence appeared to build on persistence that was reinforced by the intervention. For less experienced users, confidence seemed more closely linked to newly acquired understanding and clearer day-to-day guidance. Family and household context may also have shaped these experiences. For some participants, existing family support may have reinforced the intervention’s effects, while for others—particularly those with less immediate day-to-day support—reminders and accessible guidance may have played a more compensatory role. These observations underline that the psychological value of digital support is shaped not only by technological features but also by the life contexts in which those features are used.

### Practical Implications for Primary Care

The findings have several implications for implementation in routine primary care. First, AI-HEALS appears most suitable as an adjunct to community diabetes management rather than a replacement for clinicians. Community physicians or nurses could introduce the mini program during follow-up visits, confirm medication and monitoring schedules, and use subsequent visits to review questions or troubleshoot difficulties. Second, the predominance of lower-burden engagement suggests that pushed content and reminders may serve as the most feasible default components for routine implementation, while more interactive functions, such as the chatbot, may work better as optional layers for patients who wish to engage more actively. Third, the variation in participants’ accounts highlights the need for layered and literacy-sensitive design. Brief onboarding, plain-language instructions, and family-assisted use may be especially helpful for some users, while provider training, workflow integration, privacy procedures, and maintenance planning will be important for broader scale-up.

### Interpretive Contributions

This study contributes qualitative evidence on an AI-enabled diabetes education intervention delivered through a familiar social platform in Chinese community settings. Its main contribution lies in clarifying how participants made use of digital support in practice: through the reinforcement of awareness, the translation of advice into everyday action, selective but meaningful engagement, and increased psychological reassurance. More broadly, the findings suggest that the perceived value of AI-enabled self-management support depends not only on algorithmic tailoring but also on whether the intervention is accessible, low burden, and well aligned with users’ daily routines.

### Limitations

Several limitations should be considered when interpreting these findings. First, the 3-month follow-up captures early perceptions and short-term behavioral experiences only; it does not allow claims about long-term sustainability, and statements in this paper about maintenance should therefore be interpreted as perceived early continuation rather than established long-term adherence. Second, although 17 interviews were sufficient for thematic saturation, the sample remained small and context-specific, which limits transferability beyond Beijing community settings. Third, selection bias is possible because interviewees were drawn from the intervention arm and agreed to discuss their experiences; participants who were more willing to engage with AI-HEALS may have been more likely to participate, potentially overrepresenting favorable views. Fourth, the commercial nature of the dialog platform limited our access to detailed training data, internal validation metrics, and model specifications, constraining technical reproducibility. Fifth, social desirability bias may have influenced participants’ accounts, particularly because interviewers were linked to the broader study team. Finally, missing information on diabetes duration for some participants reduced our ability to examine duration-related patterns in depth. Taken together, these limitations indicate that the findings should be interpreted as explanatory qualitative evidence of perceived mechanisms and implementation considerations rather than definitive evidence of long-term effectiveness or generalizable user responses.

### Conclusion

This explanatory qualitative study provided insight into how patients with type 2 diabetes experienced AI-HEALS, a WeChat-based AI-enabled health education system. Participants generally perceived the intervention as helpful for strengthening awareness, supporting everyday self-management, and improving confidence, although experiences differed according to digital literacy, prior routines, and stage of illness. The findings suggest that meaningful benefits may arise even when engagement is concentrated in lower-burden features such as reminders and pushed content. At the same time, the study highlights the importance of cautious interpretation, literacy-adaptive design, and integration with routine primary care. Future studies should test the long-term sustainability of these perceived benefits and further evaluate how algorithmic tailoring, human support, and implementation context interact in larger and more diverse populations.

## Supplementary material

10.2196/84605 Multimedia Appendix 1 Detailed demographic characteristics of interviewees.

## Abbreviations

AI: artificial intelligence
AI-HEALS: Artificial Intelligence–Based Health Education Accurately Linking System
HbA1c: hemoglobin A1c
mHealth: mobile health
MTM: Multi-Theory Model
NLP: natural language processing
T2DM: type 2 diabetes mellitus
